# Machine Learning Methods for Mineralization-Based Biodegradation Prediction in Polyhydroxyalkanoate-Based Biopolymers: Insights from Lab-Scale Experiments

**DOI:** 10.3390/polym18091076

**Published:** 2026-04-29

**Authors:** Marianna I. Kotzabasaki, Leonidas Mindrinos, Nikolaos P. Sotiropoulos, Konstantina V. Filippou, Chrysanthos Maraveas

**Affiliations:** Department of Natural Resources Development and Agricultural Engineering, Agricultural University of Athens, Iera Odos 75, 11855 Athens, Greece; mariannakotz@aua.gr (M.I.K.);

**Keywords:** quantitative structure-activity relationship, polyhydroxyalkanoates, biodegradation prediction, mineralization, polymer informatics, machine learning

## Abstract

The use of bio-based and biodegradable plastic products (BBpPs) ensures the mitigation of environmental effects of fossil-based plastics, especially in humanitarian crises where waste management is challenging. Polyhydroxyalkanoates (PHAs) are promising biodegradable biopolymers that are biocompatible and do not cause microplastic pollution. However, experimental assessment of PHA biodegradation is challenged by its time- and resource-intensiveness. In this study, a comprehensive computational Quantitative Structure–Activity Relationship (QSAR)-based approach was developed to predict biodegradability of short chain length (scl)-PHA-based formulations consisting of various additives and building blocks. A novel curated dataset for the (scl)-PHA poly(-3-hydroxybutyrate-co-3-hydroxyvalerate) (PHBV), with literature-reported environmental and biodegradation parameters from lab-scale experiments in soil, marine, freshwater and compost systems, was constructed and used to develop and validate the introduced approach. Random forest (RF) and Extreme Gradient Boosting (XGBoost) machine learning (ML) models were optimized and validated with cross-validation and test set predictions. The optimal models reported high accuracy values of the coefficient of determination R^2^, indicating excellent relationships between structure and biodegradation metrics. Further analysis of descriptor variable importance confirmed that biopolymer biodegradability was favorably affected by biodegradation time, while mechanisms, environmental conditions, and additives contributed secondary yet physically consistent effects. The proposed QSAR framework demonstrated a robust and interpretable web-based tool for predicting the environmental fate of PHBV in natural environments and supported the sustainable safe-by-design (SSbD) approach of next-generation biodegradable polymers.

## 1. Introduction

PHAs, a new class of bio-based and biodegradable materials, are biosynthesized, meaning that they are accumulated by numerous bacteria as intracellular carbon and energy storage materials in the form of granules. The materials are formed when nutrients such as phosphorus and gases such as nitrogen and oxygen are limited but where carbon is available in excess. The most common types of scl-PHAs are poly(3-hydroxybutyrate) (PHB) and its copolymer PHBV [1,2,3] and present a strong potential to replace traditional plastics in different sectors.

The biodegradation efficiency of scl-PHAs is closely linked to the physical and chemical characteristics of the core polymers. Degradation rates depend on the: (i) crystallinity; (ii) copolymers; and (iii) copolymeric structure. In a PHBV copolymer, the 3-hydroxyvalerate (3-HV) insertion has a larger amorphous region that is more vulnerable to enzymatic attack. This is due to the easier water penetration, absorption, and sensitivity of the isodimorphic crystalline region to the enzyme’s catalytic region. Therefore, biodegradation varies depending on the crystallinity induced by the processing method. Further, based on the copolymer ratio, the amorphous region can be modified, and the enzymatic activity of the depolymerase can be maximized. Copolymers are known to biodegrade faster than homopolymers due to the presence of the inherent amorphous region. For example, PHBV is formed by the addition of HV to PHB, where the relative fractions of HB and HV define modulating crystallinity. This lower crystallinity of the copolymer is directly related to higher enzyme activity measured by the weight loss of PHBV. The increase in HV content decreases crystallinity while improving the biodegradation rate. Research indicates that HV content ranging 40–50% produces the fastest biodegradation rates in soil [4,5,6]. This result is also reflected in anaerobic environments.

Polymer informatics applies ML-based QSAR models to accelerate biodegradable polymers discovery and rational design prior to laboratory validation [7]. Prolonged experimental biodegradation testing times end up harming the evaluation of a new biodegradable polymeric material and delaying the development of formulation adjustments to ensure the desired level of biodegradation. There is currently only one QSAR model for predicting PHBV formulations’ biodegradation-induced weight loss under both laboratory and real environmental conditions produced by the authors [8]. Meanwhile, few ML models using QSARs exist to predict PHAs’ key physicochemical properties, including melting temperature (T_m_), and glass transition temperature (T_g_) [9,10]. The limitation is particularly pronounced for PHAs’ biodegradation modeling, where the lack of large-scale, high-quality, open-access datasets and standardized data collection methods hinders further progress. Addressing these challenges ensures: (1) creating specialized data libraries that systematically collect and organize information on the properties of scl-PHA-based materials combined with biodegradability outcomes for reliable environmental modeling and decision-making; (2) designing these libraries to be comprehensive, inclusive, and reproducible; (3) curating extensive, detailed data on physicochemical, environmental and biodegradation properties; (4) integrating data at multiple levels, including monomer composition, material performance, degradation behavior, and environmental fate, to provide a holistic view of scl-PHAs behavior; and (5) long-term usability and inclusiveness of these datasets in accordance with the FAIR principles [11].

The primary contribution of this work is to present a complete modeling workflow—from data collection to model development—to predict the biodegradation profiles of scl-PHA (PHBV)-based materials based on lab-scale experimental data simulating natural environments, with acceptable accuracy and significantly reduced evaluation time in comparison with standard experimental tests. Building on previous work [8], this study extends the application of QSAR-based ML models to predict mineralization-based biodegradation behavior in PHBV-based materials. The proposed framework provides a design-oriented computational tool to support the development and optimization of more sustainable polymeric materials.

In the present study, a novel mineralization-based biodegradation dataset of scl-PHA (PHBV)-based formulations was constructed by collecting, assembling, and curating data on their physicochemical, environmental, and biodegradation properties obtained from soil, marine, and compost environments. Subsequently, we developed and validated regression-based QSAR models for predicting degradation behavior of the studied formulations, with high accuracy values of R^2^, which indicated excellent relationships between structure and biodegradation metrics. Finally, we analyzed the most important attributes affecting the biodegradation behavior of the materials, driven by the interplay between intrinsic material properties and extrinsic physicochemical environmental factors. The RF model was implemented as a ready-to-use web application in the Jaqpot computational platform.

## 2. Materials and Methods

### 2.1. Workflow of Model Development

**Figure 1:**
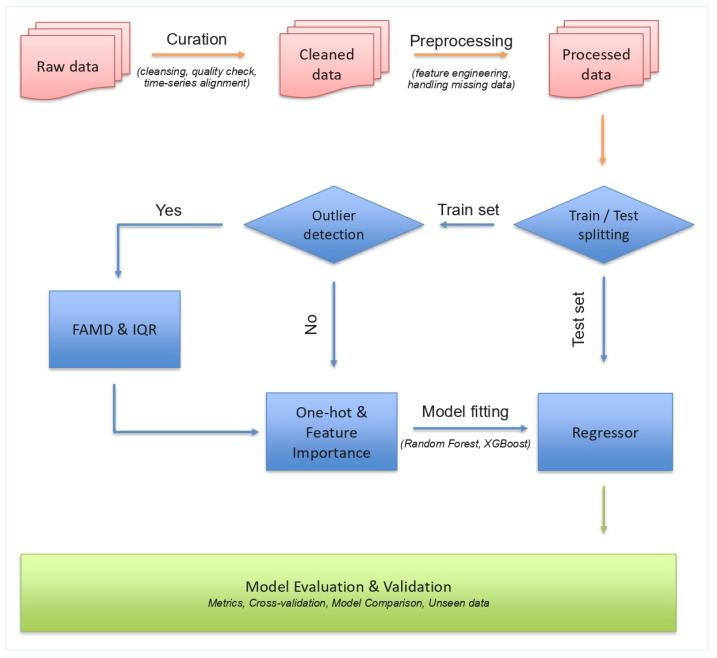
Workflow of QSAR model.

The general workflow applied in this study consists of the following: data collection, data curation, data pre-processing, model fitting, and model evaluation. The raw data were manually extracted from the literature through an initial data curation process. The next step involved data pre-processing with data normalization, feature engineering, and handling of missing values. The final processed dataset was then divided into three subsets: training, test, and validation sets. The validation set included unseen instances used to examine the model’s ability to generalize predictions. Outlier exclusion was performed on the training set using factorial analysis of mixed data (FAMD) [12] and the interquartile range (IQR) method [13]. The regression models used in this work included RF [14] and XGBoost) [15]. These are widely used regression models for polymer informatics; see [8] for a recent application on weight loss prediction of PHBV. The performance of the regression models was validated using the R^2^ values and the error metrics: root mean squared error (RMSE) and mean absolute error (MAE). The prescribed workflow is depicted in Figure 1.

### 2.2. scl-PHA-Based Formulations’ Biodegradation Database Construction

Data collection was the first stage in constructing the biodegradation database for scl-PHAs with a focus on PHBV-based formulations. The quality of the data was critical to the overall study. Data were gathered through a comprehensive literature mining process of peer-reviewed research articles, theses, and experimental studies related to PHBV degradation in different environmental conditions. The selected studies investigated the degradation behavior of the studied materials in soil, compost, freshwater, and marine environments, considering mainly controlled laboratory conditions. Studies reporting “CO_2_ evolution” as a quantitative degradation metric were identified, revealing the degree of mineralization-induced biodegradation or biodegradation percentage (“B%”, output variable) together with associated environmental and materials parameters (molecular, structural, morphological, etc.) of the studied formulations.

Data obtained through these sources were more credible and accurate because they were directly obtained from laboratory experiments. However, rich data were contained in articles, and collecting them manually was time-intensive. All the available published data, including numeric data, polymer names, and property values, were stored in a tabular format (Excel files) to ease transfer and utilization. Some of the property data were extracted from graphs and digitized tables of the available studies and Supplementary Materials. Images and graphs were quickly converted into numerical data using the professional Plotdigitizer software (version 3) [16].

#### 2.2.1. Literature Search Strategy

A systematic literature search was performed in accordance with the PRISMA 2020 guidelines using the Scopus database [17,18]. The search identified publications investigating the biodegradation properties of PHBV-based materials in the presence of natural and synthetic additives such as fillers, plasticizers, stabilizers, bio-based compounds, and other components. Scopus was selected due to its extensive coverage of the literature in material science, polymer engineering, and environmental degradation.

The search query was designed to include Boolean operators, targeting the Title, Abstract, and Keywords fields (TITLE-ABS-KEY). Three blocks of concepts were joined together using the AND operator: (i) the name of the copolymer PHBV, as the name of the biodegradable polymer of study, (ii) the biodegradation-related properties, and (iii) a targeted set of additives and natural components added to PHBV-based formulations to modulate their biodegradation behavior.

The final search string was structured as follows:

TITLE-ABS-KEY ((“PHBV” OR “polyhydroxybutyrate-co-valerate” OR “poly(3-hydroxybutyrate-co-3-hydroxyvalerate)”) AND (“biodegradation” OR “decomposition” OR “microbial degradation” OR “mineralization”) AND (“lignin” OR “lignins” OR “citric acid” OR “citric ester” OR “acetyl tributyl citrate” OR “ATBC” OR “Vish-E filler” OR “starch” OR “starch-based fillers” OR “alginate” OR “alginic acid” OR “pure cellulose” OR “wood flour” OR “woodflour” OR “WF” OR “wheat straw fibre” OR “wheat straw fiber” OR “lignocellulosic” OR “miscanthus” OR “olive pomace” OR “catechin” OR “ferulic acid” OR “vanillin” OR “polylactic acid” OR “PLA” OR “flax fibers” OR “flax fibres” OR “calcium carbonate” OR “CaCO3” OR “boron nitride” OR “quercetin” OR “DDGS” OR “distillers dried grains with solubles” OR “posidonia oceanica” OR “gallic acid” OR “ammonium quaternary salts” OR “epoxidized soybean oil” OR “castor oil” OR “limonene” OR “thymol” OR “sorbitol” OR “maltodextrin”)).

There were no restrictions on the year of publication to ensure wide historical coverage. Only English-language articles were selected. In the screening process, reviews, conference papers, editorials, notes, and book chapters were eliminated.

#### 2.2.2. Study Selection and Screening

The identified records from the database search were all exported for evaluation. The title and abstract of the identified records were assessed for relevance regarding the biodegradation of PHBV in the presence of additives in natural environments. The full-text articles were further screened based on predefined inclusion and exclusion criteria.

The inclusion criteria included studies that:

(i) Examined PHBV or PHBV-based composites;
(ii) Analyzed biodegradation, microbial degradation, or mineralization processes;
(iii) Evaluated the biodegradability using time-series measurements of biodegradation percentage over time;
(iv) Evaluated the use of additives such as fillers, plasticizers, nucleating agents, bio-derived materials, or other compounds.

The exclusion criteria eliminated studies focused on other polymers other than PHBV, non-biodegradation-based degradation mechanisms, biodegradation rates measured by other methods other than CO_2_ evolution, and additives not incorporated into the polymer matrix.

**Figure 2:**
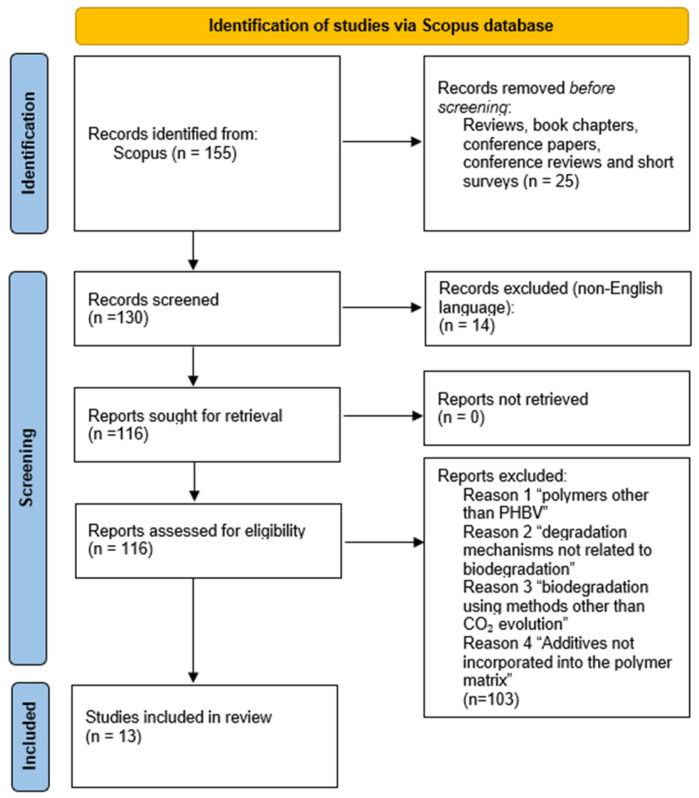
PRISMA 2020 flow diagram [18] illustrating the identification, screening, eligibility, and inclusion of studies investigating the biodegradation of PHBV-based materials containing natural and synthetic additives.

Using the targeted search query in the advanced search of Scopus, 155 results were identified for studies from 1991 to March 2025 that met the search criteria. A detailed examination of each study based on the predefined inclusion and exclusion criteria was conducted. The inclusion criteria considered studies related to the research focus on the biodegradation of PHBV formulations containing targeted additives in natural environments. Studies were also limited to those that utilized primary methods for data collection. Although excluding non-English articles may have led to missing important insights from major agricultural countries such as Japan and China, the inclusion of only English publications guaranteed reaching an international audience. Selecting only full-text studies ensured the availability of comprehensive experimental and methodological details, resulting in a final dataset comprising 13 peer-reviewed publications [19,20,21,22,23,24,25,26,27,28,29,30,31] included in the data library for the biodegradation of scl-PHA-based materials, with a specific focus on PHBV formulations incorporating a variety of additives and building blocks. The overall study selection process is summarized below using a PRISMA 2020 flow diagram [18] (Figure 2).

#### 2.2.3. Data Extraction and Synthesis

For each eligible study, the following information was extracted: PHBV compositional parameters and physicochemical properties, type and concentration of additives or natural compounds, biodegradation conditions, test environments, and reported degradation outcomes. The information was compiled qualitatively to establish the trends of biodegradation behavior linked to different additive categories. Where numerical values were unavailable, data were digitized from figures and tables.

#### 2.2.4. Data Curation Methodology

Following data extraction, curated databases were constructed in a structured spreadsheet (Microsoft Excel) and organized into interlinked worksheets to capture the complex aspects of PHA biodegradation processes. The curated database comprised the following categories: the data extracted from the examined studies were entered into a unified database including 5 worksheets, each representing a different category of data, organized as summarized in Table S1 (Supplementary Materials). A separate tab was included to provide the list of abbreviations contained in the dataset.

Each study was assigned a unique identification number (study ID). Thereafter, the necessary information from each data category was recorded in individual rows. When a single study reported multiple physicochemical characteristics or experimental conditions, separate data instances were created to represent each condition. Despite representing different experimental scenarios, all such instances retained the study ID of their respective source study to preserve traceability. In total, 13 distinct study IDs corresponding to 98 independent instances in “Worksheet_1_Materials_features” and “Worksheet_2_Environmental_features”, and 93 independent instances in “Worksheet_3_Biodegradation_features” and Worksheet_4_Biodegradation_features” were identified, collectively generating 1414 time-resolved biodegradation observations. The distribution of instances and data pairs (biodegradation time, biodegradation percentage) per study is presented in Table S2 (Supplementary Materials). It has to be noted that the study IDs are not sequential since they were extracted from a larger data library.

Tables S3–S6 in Supplementary Materials provide a detailed overview of all input (feature) and output (target) variables used to construct the PHBV-based formulations’ biodegradation data library. Table S3 reports 29 material features (12 categorical and 17 numerical) included in the PHBV biodegradation database, describing material descriptors encompassing composition, additives, molecular descriptors, physicochemical properties, physical–morphological characteristics, and surface characteristics of PHBV-based materials. Table S4 presents 26 environmental features, including 17 numerical and 9 categorical variables encompassing biochemical condition, the physical medium, temperature, pH, moisture, salinity, nutrient content, solids composition, microbial presence, and standardized testing protocols. Table S5 summarizes the biodegradation response variables included. For each feature, the table reports the feature name, a concise description, the measurement unit, the observed value range across all studies, and whether the feature is categorical or numerical. Table S6 summarizes the biodegradation time-series data. Each presented variable is described in terms of its definition, measurement unit, value range, and type (categorical or numerical). The full curated raw data library on PHBV biodegradation, including all variables across the four categories, has been uploaded to the AUA Zenodo repository [32].

The curated raw biodegradation dataset of PHBV-based materials was used as input in supervised ML models to predict degradation percentages over time. The data were organized to explicitly define input (feature)–output (target) relationships:

- Input features (independent variables) consisted of molecular, physicochemical, and environmental descriptors such as monomer composition (%HB, %HV), additive concentration, molecular weight (Mw), number-average molecular weight (Mn), the degree of crystallinity (Xc), environmental pH, temperature, oxygen availability, microbial activity indices, etc.
- Output targets (dependent variables) corresponded to degradation performance metrics, including time-dependent degradation percentages (B%) derived from reported experimental time-series data.

Biodegradation time (days) was treated as the primary temporal variable describing the kinetics of the degradation process. However, biodegradation kinetics are inherently governed by multiple interacting physicochemical and environmental parameters. To account for this multi-parameter dependence, the model incorporated these governing factors as concurrent input features alongside biodegradation time. Specifically, the dataset includes variables describing: (i) biodegradation environment (soil, compost, freshwater, marine); (ii) biodegradation conditions (aerobic/anaerobic); (iii) temperature (T_biodeg); (iv) degradation mechanism; (v) microbial community composition; (vi) sample morphology; and (vii) additive type and concentration. This multi-dimensional representation enables the model to capture the combined effects of environmental conditions, material properties, and biological activity on biodegradation trajectories.

The generated curated dataset was harmonized to ensure consistency across studies and verify the completeness of metadata. Units of measurement were converted to SI units, terminology was aligned with ISO 472:2013 polymer definitions (Plastics—Vocabulary) [33], and redundant entries were consolidated using duplicate identification based on DOI. The harmonized datasets comply with the FAIR principles [11].

#### 2.2.5. Data Pre-Processing

The “Worksheet_3_Biodegradation_features” and “Worksheet_4_Biodegradation_features” datasets were organized in a hierarchical format, comprising study- and instance-based measurements. The first five rows and first ten columns of the dataset are presented in Tables S7 and S8 in Supplementary Materials, respectively.

All biodegradation percentage values and biodegradation time points were reshaped into separate rows where each row represented a single biodegradation observation with keys “Study_id”, “Instance”, and “Sample_name”. The resulting datasets were merged into a unified “time-percentage” dataset consisting of 1414 rows (samples) and 5 columns. The first five rows of this dataset are detailed in Table S9 (Supplementary Materials).

The “properties” dataset was obtained by merging the “Worksheet_1_Materials_features” and “Worksheet_2_Environmental_features” datasets. It comprised 93 rows, corresponding to the number of independent instances, and 59 columns. However, many features were undersampled; for example, “Mw” and “PHBV_crystallinity” contained only 3 non-zero values.

The final dataset was generated by merging the “properties” and “time-percentage” datasets. After excluding columns with less than 80% completeness, the final dataset had 1414 rows (samples) and 17 columns (features), including the target variable (Biodegradation_percentage). A summary of the final dataset is presented in Table 1.

The distribution of numerical and categorical features is presented in Figures S1 and S2 (Supplementary Materials), respectively.

As shown in the top-left plot of Figure S1, the target variables had values greater than 100%. These values originated from aerobic biodegradation experiments and reflect known artifacts of CO_2_-based mineralization measurements, in which biodegradation is quantified from cumulative mineralization curves (e.g., CO_2_ evolution or oxygen consumption) and normalized to the theoretical maximum mineralization of the polymer’s carbon content. In practice, values above 100% may arise due to experimental variability, measurement uncertainty, incomplete correction for background microbial respiration and curve-fitting or extrapolation effects. Although such values are commonly reported in biodegradation studies and indicate complete or near-complete mineralization, they do not represent physically meaningful biodegradation extents for modeling purposes. These data points were therefore excluded during data curation, resulting in a final dataset of 1364 samples.

To address feature sparsity, categorical variables in the dataset with many and/or closely related labels were restructured into fewer, more informative groups. For instance, the variable “Sample_shape_morphology” initially had 10 distinct categories; some of them were mislabeled (e.g., films, film) or represented by a small number of observations (e.g., matrix). These levels were aggregated into five broader classes: films, sheets, matrix composites, particles, and pellets. Table S10 (Supplementary Materials) presents the relations between original and merged categories. A similar strategy was applied to the variables “Degradation_Enviroment”, “PHA_degrading_microbes”, and “Additive_type_1”. An overview of all variables subjected to this categorical reduction is provided in Table S11 (Supplementary Materials).

As detailed in Table 1, the numerical feature “T_biodeg” (degradation temperature) had 13.6% missing entries associated with the experiment with study_id = 5. Since degradation temperature was closely related to the degradation environment, the missing values were imputed using a context-aware approach. As such, the missing values related to soil environment were replaced with the mean temperature of observations from the environmental category “soil”.

### 2.3. Biodegradation QSAR Model Development and Validation

The dataset was split into the training, test, and validation sets to guarantee unbiased evaluation of the performance of models. Data splitting was performed before feature selection to prevent information leakage. Dimensionality reduction and outlier exclusion were conducted only on the training data. The train–test split was performed at the instance level, allowing multiple observations from the same instance to be placed in both sets.

**Figure 3:**
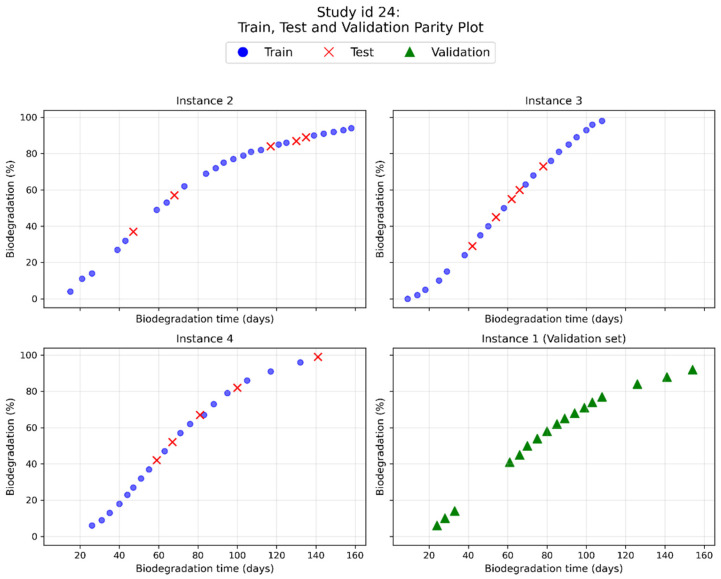
Training/test/validation splitting of the Study ID 24.

To examine generalization, a validation set was formulated by holding out entire instances. For study IDs containing more than three instances, an instance with more than five observations was randomly selected and excluded from model development. A particular case (Study_id = 24) is shown in Figure 3. This procedure resulted in a validation set of 6 full instances with 121 samples (8.9%). From the remaining 1243 data, 20% (249 samples) were used for the test set, and the remaining 80% (994 samples) were used for training.

To examine the effect of statistically extreme values while preserving potentially informative variability, FAMD [12] was used to transform both categorical and numerical features into a lower-dimensional component space. Outlier detection using the IQR method [13] was then performed on the FAMD components to evaluate whether extreme samples represented noise or meaningful structure, rather than to blindly remove significant data. After outlier removal, categorical features were then encoded using one-hot encoding to ensure compatibility with the ML algorithms. Feature selection was further analyzed using an RF-based approach to support dimensionality reduction by eliminating features with negligible importance.

RF and XGBoost regression models were assessed on the final dataset, and their performance was compared using the error metrics R^2^, RMSE, and MAE. Cross-validation and hyperparameter tuning were employed to enhance model robustness.

## 3. Results and Discussion

### 3.1. Data Curation

A structured dataset on the biodegradation of PHBV copolymers or blends formulated with a range of additives and building blocks was manually compiled via a literature data mining of 13 peer-reviewed studies, resulting in 93 independent instances and 1364 sample points. The curated raw dataset used for the data pre-processing step is publicly available in the associated data repository (AUA Zenodo repository) [32]. An overview of the compositional, molecular, physicochemical, and environmental descriptors of the PHBV-based formulations assessed across the multiple cited studies is presented in Tables S3–S6 in Supplementary Materials. The biodegradation endpoint was precisely defined in accordance with standardized biodegradability assessment practices, based on “CO_2_ evolution measured as time-series data and expressed as biodegradation percentage”. Degradation performance metrics were derived from published biodegradation percentage–time curves extracted directly from the literature using graphical data digitization.

### 3.2. QSAR Model Performance

RF and XGBoost models were initially tested on the full dataset without outlier exclusion. Both models effectively predicted biodegradation percentage, achieving R^2^ values greater than 0.98 on the training set and 0.95 on the test set. XGBoost showed slightly lower RMSE and MAE values than Random Forest on the training set, while the opposite trend was observed on the test set. The predictive performance, summarized in Table 2, indicated an excellent fit to the data and similar predictive accuracy of the two models.

To optimize predictive performance, a grid search-based hyperparameter tuning procedure was applied separately to the RF and XGBoost models. For each algorithm, a predefined range of hyperparameter values was selected. The combination of hyperparameters that minimized the cross-validated error on the training data was selected as optimal for each model. Tables S12 and S13 in Supplementary Materials provide a detailed summary of the candidate and selected hyperparameters for RF and XGBoost, respectively.

The optimized models were tested using 5-fold cross-validation, and the results are shown in Table 3. For RF, the test R^2^ decreased slightly after tuning, but cross-validation stability improved (CV mean R^2^ = 0.952, SD = 0.013), indicating more consistent performance across folds. In contrast, XGBoost showed improvement: the test R^2^ of 0.951 increased to 0.960 after tuning, reflecting enhanced generalization. Overall, both models benefited from hyperparameter tuning.

**Figure 4:**
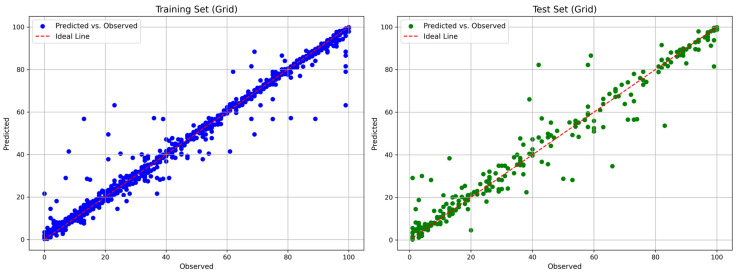
Comparison plot of the predicted and true biodegradation percentage values from the RF model.

**Figure 5:**
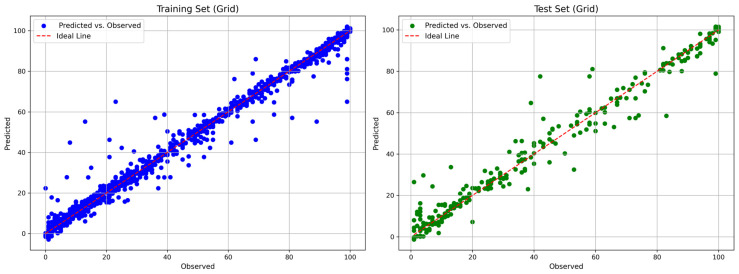
Comparison plot of the predicted and true biodegradation percentage values from the XGBoost model.

Figure 4 and Figure 5 show the comparisons between the predicted and observed biodegradation percentages of the optimized RF and XGBoost models, respectively.

**Figure 6:**
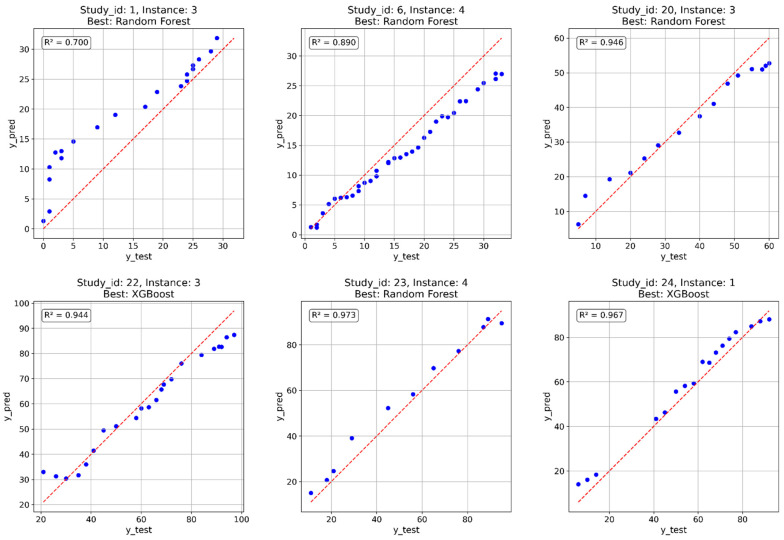
Comparison of predicted and true biodegradation percentage on fully unseen instances.

The optimized models were also tested on the validation dataset, which was not involved in either training or hyperparameter tuning. The corresponding R^2^ values are reported in Table 4. The RF model achieved an R^2^ of 0.969, indicating strong predictive accuracy on this dataset, while the XGBoost model obtained a slightly lower R^2^ of 0.956. Compared to the previous results, both models achieved high performance, confirming their ability to generalize beyond the data used for training and tuning. The comparison plots of the six totally unseen instances are presented below in Figure 6.

RF achieved the best predictions in 4 out of 6 cases. Poor predictive performance was observed for only one instance. However, the model predictions remained accurate at longer biodegradation times, which was critical for testing polymer biodegradability.

**Figure 7:**
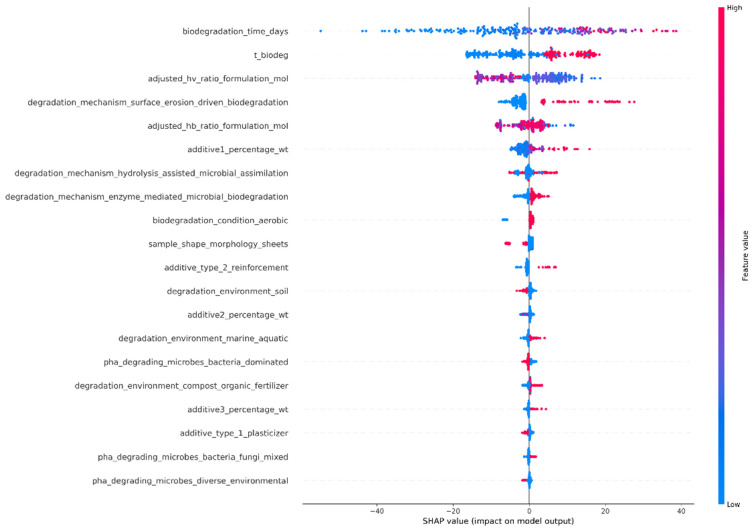
SHAP summary plot showing the impact of input features for the RF model.

**Figure 8:**
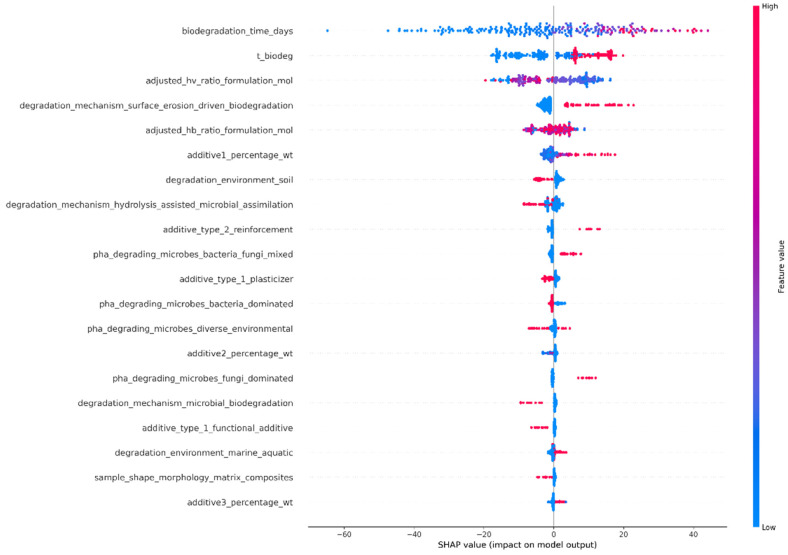
SHAP summary plot showing the impact of input features for the XGBoost model.

Feature importance was examined using SHapley Additive exPlanations (SHAP) values [34]. The contribution of each input variable to the model predictions is depicted in the SHAP summary plots. The plots for the RF and XGBoost models are shown below in Figure 7 and Figure 8, respectively. Although the two models rely on different learning mechanisms, the same set of features appeared as most influential in both cases, showing that their impact is highly independent of the ML modeling.

The SHAP summary plot in Figure 7 revealed the significance of physicochemical and environmental properties in the biodegradation process of PHBV-based materials. The highest significance was attributed to “biodegradation_time_days”. This was attributed to the inherently kinetic nature of the biodegradation process. The significant positive effect of the feature “T_biodeg” also underscored the role of temperature in enhancing the process through increased molecular mobility and reaction rates. The significant non-linear effect of compositional properties, “adjusted_hv_ratio_formulation_mol” and “adjusted_hb_ratio_formulation_mol”, underscored that the process was not controlled by a simple monotonic dependence on the composition of the copolymer. Instead, the results suggested that there was an optimal range within which the composition resulted in maximum biodegradation performance due to the interplay between crystalline and amorphous regions in the polymer, allowing for both water penetration and enzymatic accessibility.

In addition to the compositional properties, microstructural and interfacial characteristics, including additives, morphology, and degradation mechanisms, also significantly impacted the performance of PHBV-based materials. The positive additive content indicated that the material performance was improved by the presence of additives, which acted as microstructural modifiers. The surface erosion degradation mechanism had a strong positive value, indicating a significant impact of surface erosion on the degradation process of PHBV materials. The films and sheets, related to the microstructure, had a moderate value, indicating that the surface-to-volume ratio impacted the diffusion-controlled degradation process. Therefore, the degradation process of PHBV materials resulted from the combination of structure, properties, and environment, as indicated by the SHAP analysis derived for the RF model (Figure 7). The presence of soil and bacteria, which had a strong positive value, indicated that the degradation process of PHBV materials was more efficient in a soil-bacteria environment.

The dominant importance of biodegradation time reflected the kinetic nature of the process; however, its effect should not be interpreted in isolation. The SHAP analysis confirmed that biodegradation behavior was governed by the combined influence of temperature, microbial community composition, degradation mechanism, and polymer composition. These parameters modulated the rate and extent of degradation under different environmental conditions, demonstrating that the model effectively captured the multi-factorial nature of polymer biodegradation rather than a simple time-dependent trend.

Other important properties and processes were also revealed by the SHAP summary plot (Figure 8) derived for the XGBoost model. The significant positive effect of the feature “degradation_mechanism_surface_erosion_driven_biodegradation” indicated that surface-controlled degradation processes were dominant in determining the biodegradation rates. The significant positive effect of additive-related properties, such as “additive1_percentage_wt”, revealed that these properties affected the biodegradation process by influencing the microstructure of the polymer, potentially increasing the hydrophilicity. The fact that microbial activity, especially in bacteria-dominated systems, resulted in increased biodegradation rates emphasized the biological aspect of the process. The morphological properties and environmental properties had lower significance in determining the biodegradation rates. The results obtained from the SHAP summary plot for the XGBoost model exhibited that the biodegradation process of PHBV-based materials was controlled by a complex interplay between molecular composition, degradation mechanisms, and biologically mediated environmental properties.

**Figure 9:**
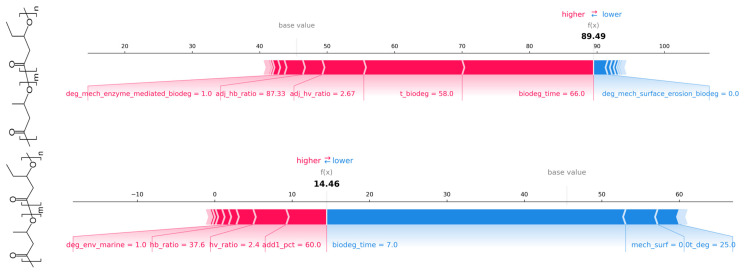
SHAP force plot explaining the RF regression prediction of biodegradation percentage for a single test observation: study_id = 16, instance = 3 (**top**) and study_id = 5, instance = 31 (**bottom**).

The SHAP force plot (Figure 9) presents the prediction of the biodegradation percentage of two individual test observations using the Random Forest model. The prediction process began with the model’s base value, and the individual effects of each input variable were afterwards incorporated. In the force plot visualization, variables highlighted in red contributed to an increase in the predicted value, whereas those shown in blue contributed to a decrease. The length of each bar indicates the relative strength of that variable’s influence on the final prediction.

The effect of outlier removal on model robustness was investigated for the optimized RF and XGBoost models. Predictive performance was compared between models trained on the full dataset and those trained after statistical outlier exclusion using the IQR method. A total of 75 samples (7.5%) were removed from the training set of 994 observations.

The resulting performance metrics are summarized above in Table 5. For both RF and XGBoost, excluding outliers led to a consistent decrease in predictive accuracy across test and unseen datasets, with a higher impact observed for Random Forest. The results indicated that the so-called “outliers” are not noise but instead carried physically relevant information describing extreme but valid biodegradation conditions, which are important for model generalization in real environmental scenarios. This conclusion was further supported by the Williams plot (Figure S4 in Supplementary Materials), which indicated that all samples were placed within the acceptable leverage and standardized residual limits, confirming the absence of influential outliers in the dataset. Similar observations have been reported in other polymer informatics studies [8,35,36]. In the Supplementary Materials, Table S14 outlines the DOA of the model by defining the ranges of the input numerical features.

### 3.3. Web Implementation of the Model

The source code for developing the model is available at: https://github.com/FSL-AUA/Biodegradation-model.

The model has been implemented as a web service in the Jaqpot 5 modeling platform (https://app.jaqpot.org/) and is available at the following URL: https://app.jaqpot.org/dashboard/models/2346/description.

Prior to deployment, the model was evaluated through a comprehensive applicability domain (AD) analysis to ensure that new input samples fall within the descriptor space defined by the training data. Both the leverage and bounding box methods were applied to characterize the model’s AD, allowing users to verify whether selected input feature values lie within the reliable prediction domain of the RF model.

To access the model, the interested user should first register on the Jaqpot 5 web platform. Detailed instructions on how to use the web application and generate predictions are provided in the Supplementary Materials.

## 4. Conclusions

A QSAR-based ML framework was developed to predict the mineralization-based biodegradation of PHBV-based formulations, drawing on a curated dataset of 93 experimental instances and 1364 data pairs compiled from 13 peer-reviewed studies spanning soil, marine, freshwater, and compost environments. RF and XGBoost regression models were trained, cross-validated, and evaluated on independent validation instances, achieving test-set R^2^ of 0.95 and 0.96, cross-validated mean R^2^ exceeding 0.95, and validation-set R^2^ of 0.97 and 0.96, respectively; uncertainty is higher for formulations outside the defined applicability domain or involving underrepresented additive–environment combinations. SHAP analysis identified biodegradation time as the dominant predictor, consistent with the kinetic nature of the process, with secondary contributions from temperature, microbial community type, PHBV copolymer composition (non-linear HV/HB ratio effect), surface-erosion degradation mechanism, and additive-induced microstructural modifications. Outlier analysis confirmed that statistically flagged samples encoded physically meaningful extreme conditions rather than noise, as their removal consistently reduced model performance, and the Williams plot verified that all observations lied within acceptable leverage and residual limits. The RF model was deployed as an open-access web tool on the Jaqpot platform, supporting SSbD strategies for next-generation biopolymers. Future work should focus on integrating the existing dataset with experimental biodegradation data across different environments (e.g., field, soil and marine) alongside the incorporation of additional physicochemical descriptors such as crystallinity, molecular weight and additive-based descriptors (see Table S15 in the Supplementary Materials) to further improve robustness, generalizability and applicability.

## Figures and Tables

**Table 1:** Feature and type summary of the final dataset.

Column	Completeness	Type
Biodegradation_time_days	100%	Numerical
Biodegradation_percentage	100%	Numerical
Adjusted_HB_ratio_formulation (mol%)	100%	Numerical
Adjusted_HV_ratio_formulation (mol%)	100%	Numerical
Biodegradation_condition	100%	Categorical
Degradation_mechanism	100%	Categorical
T_biodeg	86.4%	Numerical
Biodegradation_environment	100%	Categorical
Additive_type_1	100%	Categorical
Additive1_percentage (wt%)	100%	Numerical
Additive_type_2	100%	Categorical
Additive2_percentage (wt%)	100%	Numerical
Additive_type_3	100%	Categorical
Additive3_percentage (wt%)	100%	Numerical
PHA_degrading_microbes	97.2%	Categorical
Sample_shape_morphology	100%	Categorical
Additives	100%	Categorical

**Table 2:** Model performance on training and test sets.

Model	Set	R^2^	RMSE	MAE
Random Forest	Training	0.983	4.37	1.87
	Test	0.947	7.53	4.19
XGBoost	Training	0.982	4.49	2.17
	Test	0.951	7.29	3.99

**Table 3:** Performance of optimized models.

Metric	Random Forest	XGBoost
Train R^2^	0.983	0.980
Test R^2^	0.947	0.959
CV Mean R^2^	0.952	0.960
CV SD R^2^	0.013	0.013

**Table 4:** Model performance on the validation set (unseen instances).

Model	R^2^ (Unseen Dataset)
Random Forest	0.967
XGBoost	0.956

**Table 5:** Outlier exclusion effect on model performance.

Model	Outlier Exclusion	Train R^2^	Test R^2^	CV Mean R^2^	CV SD R^2^	Unseen R^2^
Random Forest	No	0.983	0.950	0.953	0.013	0.958
	Yes	0.980	0.860	0.944	0.018	0.678
XGBoost	No	0.980	0.960	0.960	0.014	0.941
	Yes	0.978	0.838	0.949	0.016	0.716

## Data Availability

The datasets used for training and evaluating the ML models were derived from curated biodegradation data reported in the literature and compiled within the framework of this study. The implementation of the ML models, including data pre-processing, feature selection, model training, and evaluation scripts, is publicly available via GitHub at: https://github.com/FSL-AUA/Biodegradation-model. In addition, the trained models and their associated feature sets are accessible through the Jaqpot platform: Biodegradation percentage regression model A.N.I.P.H. (Jaqpot ID: 2346): https://app.jaqpot.org/dashboard/models/2346/description.

## Supplementary Materials

The following supporting information can be downloaded at: https://www.mdpi.com/article/10.3390/polym18091076/s1.

## Abbreviations

The following abbreviations are used in this manuscript:

BBpPs	Biobased and biodegradable plastic products
PHAs	Polyhydroxyalkanoates
scl-PHAs	Short chain length polyhydroxyalkanoates
PHB	Poly(3-hydroxybutyrate)
PHBV	Poly(3-hydroxybutyrate-co-3-hydroxyvalerate)
HV	Hydroxyvalerate
HB	Hydroxybutyrate
PP	Polypropylene
PET	Polyethylene terephthalate
PE	Polyethylene
PVC	Polyvinyl chloride
QSAR	Quantitative Structure–Activity Relationship
ML	Machine Learning
RF	Random Forest
XGBoost	Extreme Gradient Boosting
R^2^	Coefficient of determination
RMSE	Root Mean Squared Error
MAE	Mean Absolute Error
SHAP	SHapley Additive exPlanations
DOA	Domain of Applicability
FAMD	Factorial Analysis of Mixed Data
IQR	Interquartile Range
CO_2_	Carbon dioxide
B%	Biodegradation percentage
Mw	Weight-average molecular weight
Mn	Number-average molecular weight
Xc	Degree of crystallinity
Tm	Melting temperature
Tg	Glass transition temperature
FAIR	Findable, Accessible, Interoperable, Reusable (principles)
PRISMA	Preferred Reporting Items for Systematic Reviews and Meta-Analyses
TITLE-ABS-KEY	Title–Abstract–Keywords
ATBC	Acetyl tributyl citrate
WF	Wood flour
DDGS	Distillers dried grains with solubles
PLA	Polylactic acid
CaCO_3_	Calcium carbonate
ASTM	American Society for Testing and Materials
ISO	International Organization for Standardization
TDS	Total dissolved solids
VS	Volatile solids
TOCA	Total organic carbon availability
TNA	Total nitrogen availability
C/N ratio	Carbon-to-nitrogen ratio
SSbD	Safe-and-Sustainable-by-Design
DATs	Digital Agricultural Technologies
mcl-PHAs	Medium chain length polyhydroxyalkanoates
CV	Cross-validation
SD	Standard deviation
SI	International System of Units
DOI	Digital Object Identifier
