# Supplementary material for: Machine Learning Methods for Mineralization-Based Biodegradation Prediction in Polyhydroxyalkanoate-Based Biopolymers: Insights from Lab-Scale Experiments
Source: Polymers (Basel). 2026 Apr 29;18(9):1076. doi: 10.3390/polym18091076 (PMC13165325; doi:10.3390/polym18091076)
Supplement: Supplementary file 1 [file polymers-18-01076-s001.zip › polymers-4270052-supplementary.pdf]

Supplementary Materials

Machine Learning Methods for Mineralization-Based Biodegradation Prediction in Polyhydroxyalkanoate-Based Biopolymers: Insights from Lab-Scale Experiments

Marianna I. Kotzabasaki, Leonidas Mindrinos, Nikolaos P. Sotiropoulos, Konstantina V. Filippou and Chrysanthos Maraveas *

Department of Natural Resources Development and Agricultural Engineering, Agricultural University of Athens, Iera Odos 75, 11855 Athens, Greece; mariannakotz@aua.gr (M.I.K.); leonidas.mindrinos@aua.gr (L.M.); nikos.sotiropoulos@aua.gr (N.P.S.); filippoukonstandina@gmail.com (K.V.F.)

* Correspondence: maraveas@aua.gr; Tel.: +30-6973739283

2. Materials and Methods

<u>Data curation</u>

Table S1. Each of the 5 worksheets represented a different category of data for the PHBV-based biodegradation data library.

Worksheets	Parameters
Worksheet_1_Materials_features	Composition details, Molecular weight distributions (Mw and Mn), Crystallinity indices, Additive presence, Respective additive concentrations, etc.
Worksheet_2_Environmental_features	Temperature, pH, Moisture levels, Oxygen availability, Microbial activity, etc.
Worksheet_3_Biodegradation_features	Degradation percentages
Worksheet_4_Biodegradation_features	Degradation time points
Worksheet_5_Metadata	Study _id, Title, Doi

Table S2. Distribution of Instances and data pairs (biodegradation time, biodegradation percentage) across studies.

Study id	No. of instances	No. of data pairs	Reference
1	4	92	[19]
2	3	79	[20]
3	3	42	[21]

4	4	98	[22]
5	48	192	[23]
6	4	130	[24]
7	3	77	[25]
15	3	171	[26]
16	4	182	[27]
20	4	59	[28]
22	5	109	[29]
23	4	81	[30]
24	4	102	[31]
**Total**	**93**	**1414**	

Table S3. Overview of materials descriptors (independent variables) of the PHBV-based biodegradation database**.**

Feature name	Description	Unit	Range	Type	Missing Percentage
Sample_name	Identifier of the PHBV-based sample or formulation	–	–	Categorical	0
Sample ratio wt/wt%	Weight-to-weight ratio of components in the formulation	–	–	Categorical	0
Monomer_A	Primary monomer composing the PHBV copolymer	–	–	Categorical	0
Monomer_B	Secondary monomer composing the PHBV copolymer	–	–	Categorical	0
Adjusted_HB_ratio_formulation	Adjusted molar fraction of hydroxybutyrate units	mol%	23.8-98.5	Numerical	0
Adjusted_HV_ratio_formulation	Adjusted molar fraction of hydroxyvalerate units	mol%	0.5-10.8	Numerical	0
Additives	Presence of additives in the formulation	–	–	Categorical	0

Additive1_name	Name of the first additive	–	–	Categorical	12.24
Additive_type_1	Type of the first additive	–	–	Categorical	13.27
Additive1_percentage	Weight fraction of the first additive	wt%	0.5 – 70	Numerical	0
Additive2_name	Name of the second additive	–	–	Categorical	73.47
Additive_type_2	Type of the second additive	–	–	Categorical	73.47
Additive2_percentage	Weight fraction of the second additive	wt%	1 – 70	Numerical	12.24
Additive3_name	Name of the third additive	–	–	Categorical	88.78
Additive_type_3	Type of the third additive	–	–	Categorical	84.69
Additive3_percentage	Weight fraction of the third additive	wt%	1 – 30	Numerical	22.45
PHBV_weight_percentage_final_formulation	PHBV content in the final formulation	wt%	27 – 100	Numerical	0
Mw	Weight-average molecular weight	kDa	400 – 600	Numerical	96.94
Density	Density of PHBV-based material	g/cm^3	0.974 – 1.56	Numerical	38.78
Water_absorption_capacity	Water absorption capacity	%	0.7-33	Numerical	51.02
Film_solubility	Solubility of PHBV films	%	1-19	Numerical	51.02
PHBV_crystallinity	Degree of crystallinity of PHBV	%	52 – 55.9	Numerical	96.94
Static_water_contact_angle	Static water contact angle indicating hydrophilicity	deg	37.3-70.1	Numerical	89.80
Sample_shape/Morphology	Physical shape or morphology of the PHBV-based sample (e.g. film, pellet, fiber, composite)	-	-	Categorical	0
Size_diameter	Diameter of the PHBV sample or	cm	0.1-9	Numerical	48.98

	morphological element (e.g., particle, sphere or cylindrical structure), as reported in the source studies				
Size_length	Length of the PHBV sample or morphological element (e.g., films or tubes), as reported in the source studies	mm	0.018-100	Numerical	83.67
Size_Width	Width of the PHBV sample or morphological element (e.g. films), as reported in the source studies	mm	10-105	Numerical	85.71
Size_Thickness	Thickness of the PHBV sample or morphological element (e.g., films), as reported in the source studies	mm	0.01-2.3	Numerical	21.43
Permeability_water_vapor	Water vapor permeability of the PHBV-based material, quantifying the rate of water vapor transmission through the sample under defined conditions	$\times 10^{-13}$ mol·m·m^{-2}·s^{-1}·Pa^{-1}	4.3-21.1	Numerical	96.94

Table S4. Overview of environmental and experimental features (independent variables) of the PHBV-based biodegradation database.

**Feature name**	**Description**	**Unit**	**Range**	**Type**	**Missing Percentage**
Sample_name	Identifier of the PHBV-based sample tested	–	–	Categorical	0
Parameter_evaluated	Environmental parameter evaluated in the study	–	–	Categorical	50
Biodegradation_condition	General biodegradation condition (e.g., aerobic, anaerobic) soil, marine, compost)	–	–	Categorical	1.02
Degradation_mechanism	Dominant biodegradation mechanism reported	–	–	Categorical	0
T_biodeg	Temperature during biodegradation experiment	°C	23 – 58	Numerical	50
T_biodeg_units	Unit used to report biodegradation temperature	–	–	Categorical	50
T_biodeg_winter_marine	Marine biodegradation temperature under winter conditions	°C	23.55	Numerical	94.9

T_biodeg_summer_marine	Marine biodegradation temperature under summer conditions	°C	23.55	Numerical	94.9
TH20_winter_marine	Water exposure time (T_{h20}) in winter marine conditions	–	23.55	Numerical	94.9
TH20_summer_marine	Water exposure time (T_{h20}) in summer marine conditions	–	23.55	Numerical	94.9
Water_pH_marine	pH of marine water during biodegradation	–	7.2 – 8.06	Numerical	87.76
Salinity_marine	Salinity of the marine environment	ppt	31.9 – 37	Numerical	87.76
Soil_moisture	Moisture content of soil	%	80 – 100	Numerical	88.78
Soil_pH	pH of soil during biodegradation	–	6.8 – 7.2	Numerical	90.82
Soil_T	Soil temperature during biodegradation	°C	24 – 28	Numerical	90.82
Compost_moisture	Moisture content of compost	%	32 – 55	Numerical	79.59
Compost_pH	pH of compost environment	–	6.5 – 8.5	Numerical	84.69
Compost_T	Compost temperature during biodegradation	°C	28 – 58	Numerical	79.59
TOCA	Total organic carbon availability	–	0.13 – 59.7	Numerical	74.49
TNA	Total nitrogen availability	–	0.12 – 0.9	Numerical	92.86
C_N_ratio	Carbon-to-nitrogen ratio of the environment	–	11.8 – 550	Numerical	86,73
TDS	Total dissolved solids content of the biodegradation environment	%	53.2-80.09	Numerical	89.80
VS	Volatile solids fraction of the biodegradation environment	%	26.6-53	Numerical	88.78
PHA_degrading_microbes	Presence or abundance of PHA-degrading microorganisms	–	–	Categorical	3.06
Degradation_Environment	Classified degradation environment	–	–	Categorical	1.02
ASTM/ISO	Standard used for biodegradation testing	–	–	Categorical	50

Table S5. Overview of materials descriptors (independent variables) of the PHBV-based biodegradation database.

**Feature name**	**Description**	**Unit**	**Range**	**Type**
Sample_name	Identifier of the PHBV-based sample	–	–	Categorical

Biodegradation_percentage	Percentage of biodegradation measured at a given time point	%	0-100	Numerical

Table S6. Overview of time-point features of the PHBV-based biodegradation database.

Feature name	Description	Unit	Range	Type
Sample_name	Identifier of the PHBV-based sample	–	–	Categorical
Biodegradation_time	Time at which biodegradation was measured	days	1 – 452.12392	Numerical

Data pre-processing

Table S7. The first 5 rows and 10 columns of the "Worksheet_3_Biodegradation_features" dataset.

Study_id	Instance	Sample_name	Biodegradation_percentage %						
1	1	PHBV	3,639	4,968	5,531	6,221	6,386	7,715	15,284
1	2	PLA/PHBV	5,386	6,841	8,151	11,063	14,556	17,176	19,651
1	3	PHBV	0,987	1,096	1,316	1,425	2,193	3,070	3,838
1	4	PLA/PHBV	0,987	1,096	1,316	1,425	2,193	3,070	5,592
2	1	alginic acid-treated flax/PHBV	5,944	10,996	15,750	18,722	25,706	27,637	29,123

Table S8. The first 5 rows and 10 columns of the "Worksheet_4_ Biodegradation_features" dataset.

Study_id	Instance	Sample_name	Biodegradation_time (days)						
1	1	PHBV	5,029	8,704	12,959	18,569	22,437	30,000	33,849
2	2	PLA/PHBV	3,868	7,157	11,025	14,700	20,309	30,000	33,269
3	3	PHBV	5,656	11,893	19,144	25,816	30,000	39,884	47,861
4	4	PLA/PHBV	5,656	11,893	19,144	25,816	30,000	39,884	48,441
2	1	alginic acid-treated flax/PHBV	1,996	4,291	5,090	6,088	7,086	8,184	9,182

Table S9. The first 5 rows of the melted and merged "time-percentage" dataset.

Study_id	Instance	Sample_name	Biodegradation_time (days)	Biodegradation_percentage %

4	5	PHBV	5,029	3,639
4	5	PHBV	8,704	4,968
4	5	PHBV	12,959	5,531
4	5	PHBV	18,569	6,221
4	5	PHBV	22,437	6,386

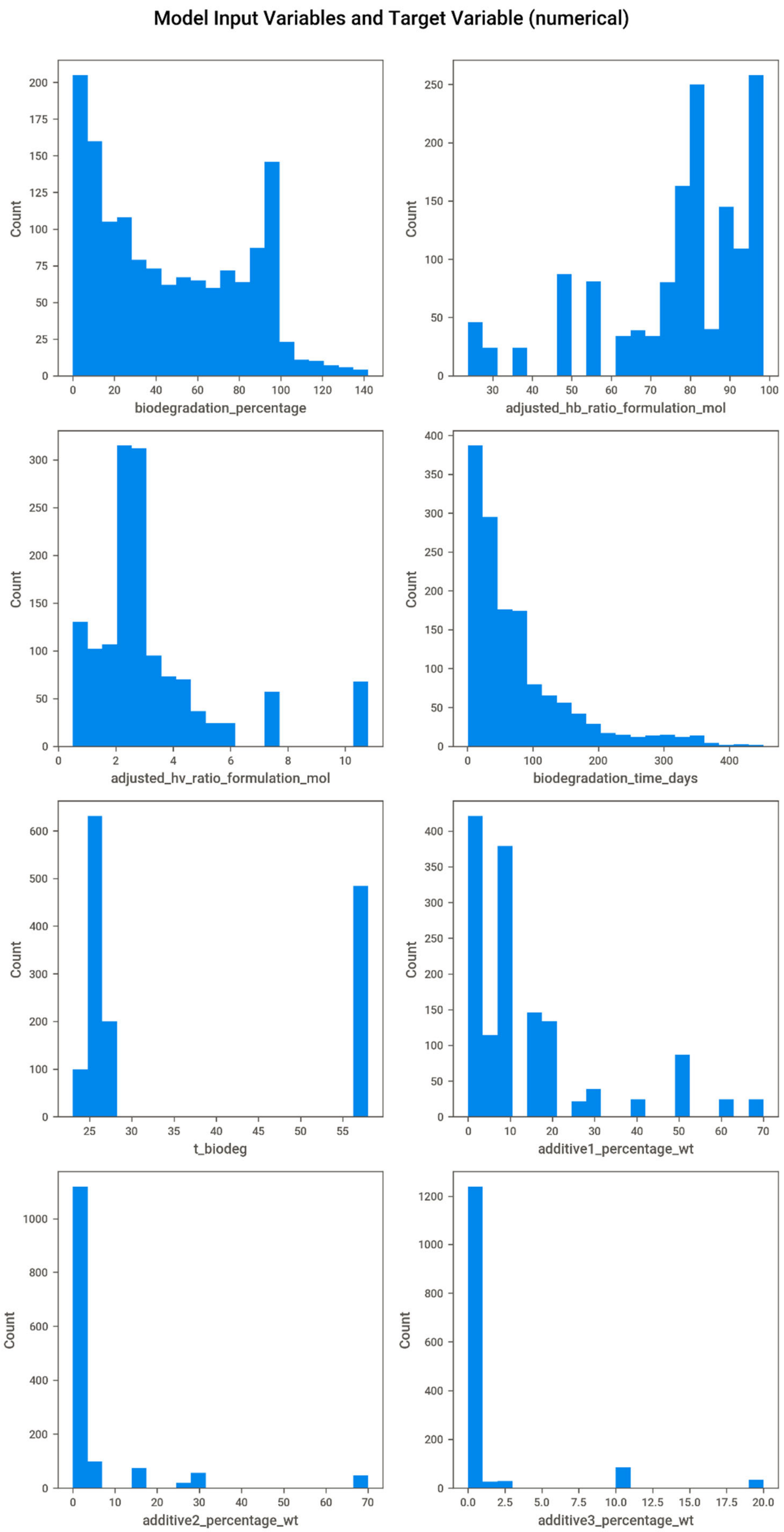

Figure S1. Distribution of numerical features and the target value "Biodegration_percentage".

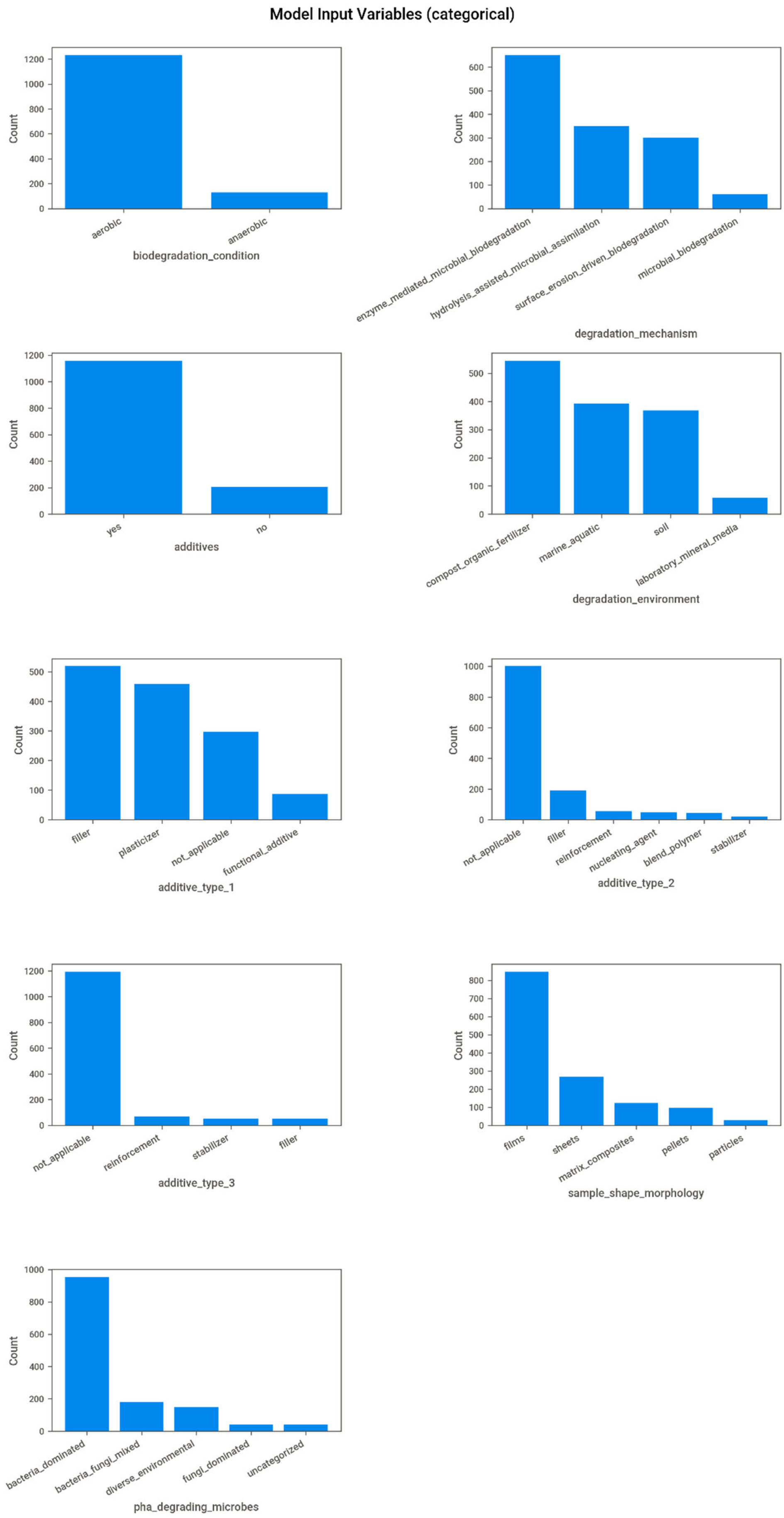

Figure S2. Distribution of categorical features.

Table S10. Category Grouping of the feature "Sample_shape_morphology".

Original Category	Count	Merged Category	Count
Films	532	films	897
circular_blend_films	192	films	
Film	92	films	
square_films	81	films	
Sheets	138	sheets	268
rectangu-lar_strips_flat_sheets	130	sheets	
continuous_phbv_ma-trix_with_dis-persed_filler_particles	109	matrix_composites	123
Matrix	14	matrix_composites	
Particle	28	particles	28
filaments_pellets	98	pellets	98

Table S11. Summary of Category Grouping of the categorical features.

Feature	No. of initial Categories	No. of merged Categories
Degradation_Environment	7	4
Additive_type_1	5	4
Sample_shape_morphology	10	5
PHA_degrading_microbes	18	5

3. Results & Discussion

3.2 QSAR model performance

Table S12. Hyperparameter grid and optimal values for the RF model.

Hyperparameter	Candidate Values	Optimal Value
n_estimators	[50, 100, 200]	200
max_depth	[10, 20, 30]	20
min_samples_split	[2, 5, 10]	2
min_samples_leaf	[1, 2, 4]	1

Table S13. Hyperparameter grid and optimal values for the XGBoost model.

Hyperparameter	Candidate Values	Optimal Value
n_estimators	[200, 400, 800]	800
max_depth	[4, 6, 8]	4
learning_rate	[0.01, 0.05]	0.05
subsample	[0.7, 0.8, 1]	1
colsample_bytree	[0.7, 0.8]	0.7

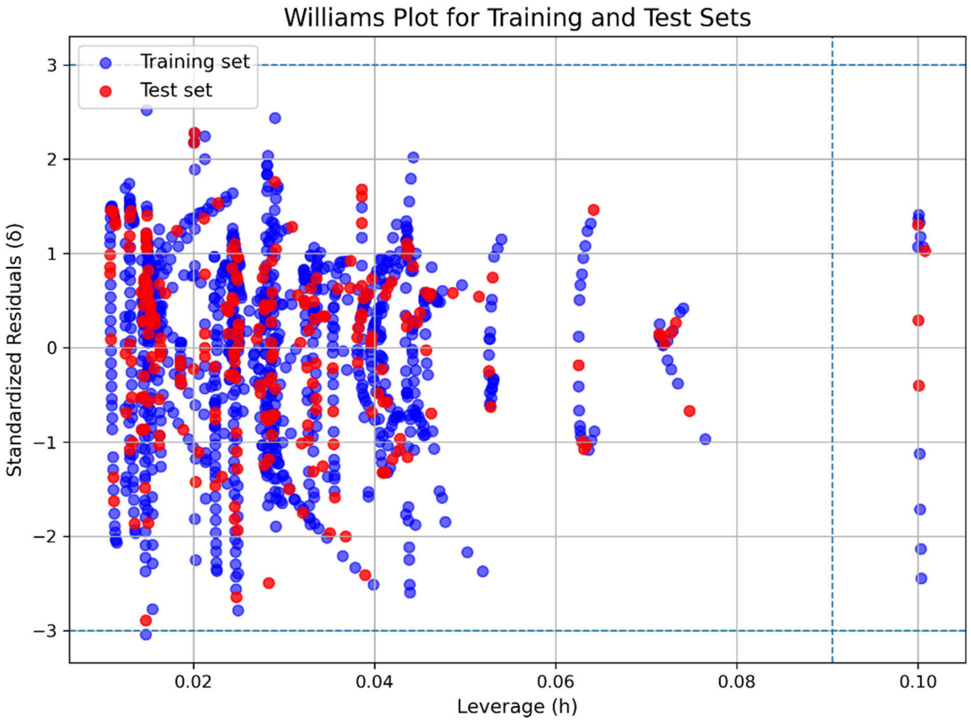

Figure S3. William's plot for the training (blue) and test (red) datasets. The dashed horizontal lines represented δ = ±3, and the vertical dashed line indicated the warning leverage threshold (h*=0.0905).

Table S14. Ranges of the numerical input features used for model development.

Feature name	Unit	Range
Additive1_percentage	wt%	0 - 70
Additive2_percentage	wt%	0 - 70
Additive3_percentage	wt%	0 - 20

Adjusted_HB_ratio_formulation	mol%	23.8 – 98
Adjusted_HV_ratio_formulation	mol%	0.5 – 10.8
T_biodeg	°C	23 - 58
Biodegradation_time_days	days	1 - 452

Table S15. Overview of major additives incorporated in PHBV-based formulations covered by the biodegradation dataset: CAS registry numbers, molecular formulae, purity grade (where reported), and key physicochemical properties relevant to biodegradation.

Additive Name	Additive Category	CAS Registry No.	Molecular Formula	Molecular Weight (g/mol)	Purity Grade (if reported in source)	Key Property Relevant to PHBV Biodegradation	Representative Source Study (Ref.)
**Fillers / Reinforcements**							
**Wood flour (WF)**	Lignocellulosic filler	N/A (mixture)	Cellulose / Hemicellulose / Lignin	~170–200 (cellulose repeat unit)	Not specified	Lignocellulosic structure increases hydrophilicity and surface roughness of PHBV matrix, enhancing water uptake and accelerating enzymatic surface attack; lignin fraction modulates microbial colonisation rate.	[24, 26]
**Wheat straw fibre**	Lignocellulosic filler	N/A (mixture)	Cellulose / Hemicellulose / Lignin	~162 (anhydroglucose repeat unit)	Not specified	Hydrophilic lignocellulosic fibres increase moisture absorption of PHBV composites, promoting ester-bond hydrolysis and microbial depolymerisation.	[28]
**Posidonia oceanica fibres**	Marine lignocellulosic filler	N/A (natural material)	Cellulose / Hemicellulose / Lignin / Phenolics	Variable	Not specified	Marine-origin fibres introduce salt-tolerant microorganisms at the fibre–matrix interface, promoting faster PHBV surface erosion in marine environments.	[22]
**Alginic acid-treated flax fibres**	Surface-treated natural fibre	9005-32-7 (alginic acid)	$C_6H_8O_6$ (alginate repeat unit)	176.12 (alginate monomer)	Not specified	Alginic acid surface treatment increases hydrophilicity of flax fibres, improving interfacial compatibility	[19, 20]

						with PHBV and creating preferential pathways for water diffusion and enzymatic attack.	
**Miscanthus fibres**	Lignocellulosic filler	N/A (mixture)	Cellulose / Hemicellulose / Lignin	Variable	Not specified	High cellulose content and porous morphology promote moisture uptake in PHBV composites; lignin component can retard enzymatic degradation at high concentrations.	[21]
**Plasticisers**							
**Acetyl tributyl citrate (ATBC)**	Citric acid ester plasticiser	77-90-7	$C_{20}H_{34}O_8$	402.47	≥99% (technical grade, where stated)	Reduces PHBV crystallinity and glass-transition temperature by increasing chain mobility; lower crystallinity expands the amorphous region accessible to enzymatic attack, accelerating biodegradation.	[27]
**Vanillin**	biobased plasticiser	121-33-5	$C_8H_8O_3$	152.15	Not specified	Vanillin (4-hydroxy-3-methoxybenzaldehyde) acts as a degradation accelerator when incorporated into poly(3-hydroxybutyrate-co-3-hydroxyvalerate) (PHBV) polymer films, enhancing the biodegradation rate. It achieves this by functioning as a plasticizing agent within the polymer matrix, which weakens the amorphous phase and makes it more accessible to enzymatic attack.	[31]
**Polyphenols / Functional Additives/Stabilizers**							

**Quercetin**	Flavonoid polyphenol	117-39-5	$C_{15}H_{10}O_7$	302.24	≥95% (HPLC, where stated)	Antioxidant activity may retard oxidative chain scission; hydroxyl groups can interact with PHBV ester bonds and modulate water sorption at the surface, affecting enzymatic accessibility.	[25]
**Gallic acid**	Phenolic acid	149-91-7	$C_7H_6O_5$	170.12	≥97% (where stated)	High polyphenol content and hydrophilicity enhance water sorption at the PHBV interface; antimicrobial properties at elevated concentrations may partially inhibit PHA-depolymerase activity.	[25]
**Ferulic acid**	Hydroxycinnamic acid	1135-24-6	$C_{10}H_{10}O_4$	194.18	Not specified	Phenolic acid that can covalently integrate into the PHBV matrix via esterification, modifying hydrophilicity and enzymatic accessibility of the polymer surface.	[21]
**Catechin**	Flavan-3-ol polyphenol	154-23-4	$C_{15}H_{14}O_6$	290.27	Not specified	Polyphenolic structure with multiple –OH groups; increases surface hydrophilicity of PHBV and may act as a substrate or inhibitor for PHA-degrading microorganisms depending on concentration.	[21]
**Biopolymer Blends**							

**Polylactic acid (PLA)**	Biodegradable polyester blend component	26100-51-6	$–[C_3H_4O_2]_n–$	72.06 (repeat unit)	Not specified	PLA/PHBV blending alters crystallinity and creates a biphasic morphology; PLA-rich domains biodegrade more slowly than PHBV under soil/compost conditions, modulating overall composite mineralisation rate.	[19, 20]
**Alginic acid (alginate)**	Polysaccharide biopolymer additive	9005-32-7	$–[C_6H_7O_6Na]_n–$	~198 (sodium alginate repeat unit)	Not specified	Hydrophilic polysaccharide that increases water uptake capacity of PHBV films; rapid dissolution in aquatic and compost environments creates porosity, enhancing microbial colonisation and enzymatic attack.	[19, 20]
**Inorganic Fillers**							
**Calcium carbonate ($CaCO_3$)**	Inorganic mineral filler	471-34-1	$CaCO_3$	100.09	Not specified	Inert filler that increases surface roughness and creates microvoids at the filler–matrix interface; slightly alkaline hydrolysis products ($Ca(OH)_2$, CO_2) may locally elevate pH and promote ester-bond hydrolysis in PHBV.	[29]
**Agro-industrial By-products**							
**Olive pomace**	Agro-industrial by-product filler	N/A (mixture)	Cellulose / Hemicellulose	Variable	Not specified	Complex lignocellulosic and phenolic composition; hydrophilic fractions accelerate PHBV surface wetting while residual phenolics may	[30]

			/ Lignin / Phenolics / Lipids			partially inhibit microbial activity at high loading.	
**Distillers dried grains with solubles (DDGS)**	Agro-industrial by-product filler	N/A (mixture)	Protein / Starch / Cellulose / Lipids	Variable	Not specified	Protein and starch fractions are readily biodegradable; their preferential microbial consumption creates micro-porosity in the PHBV matrix, accelerating subsequent PHA depolymerase access.	[24]

Notes: Additive characterization data compiled from source publications [19–31] and standard chemical databases (PubChem, ChemSpider). CAS registry numbers sourced from PubChem (https://pubchem.ncbi.nlm.nih.gov/). N/A = not applicable (natural mixture without single CAS number). Purity grades are reported only where explicitly stated in the original source publication; absence of a value reflects the scope of the available primary literature. MW values given for the dominant repeat unit or monomer unless otherwise stated.

3.3 Web implementation of the model

Jaqpot is a cloud-based platform for deploying and sharing predictive models via web services. It supports machine learning and deep learning models and provides a user-friendly graphical interface that enables clear model documentation and interaction.

In this study, the biodegradation percentage prediction model is available at https://app.jaqpot.org/dashboard/models/2346/description

Access is provided via the Jaqpot login interface using Google or GitHub accounts or by registration and then verification is needed (see **Figure S4**).

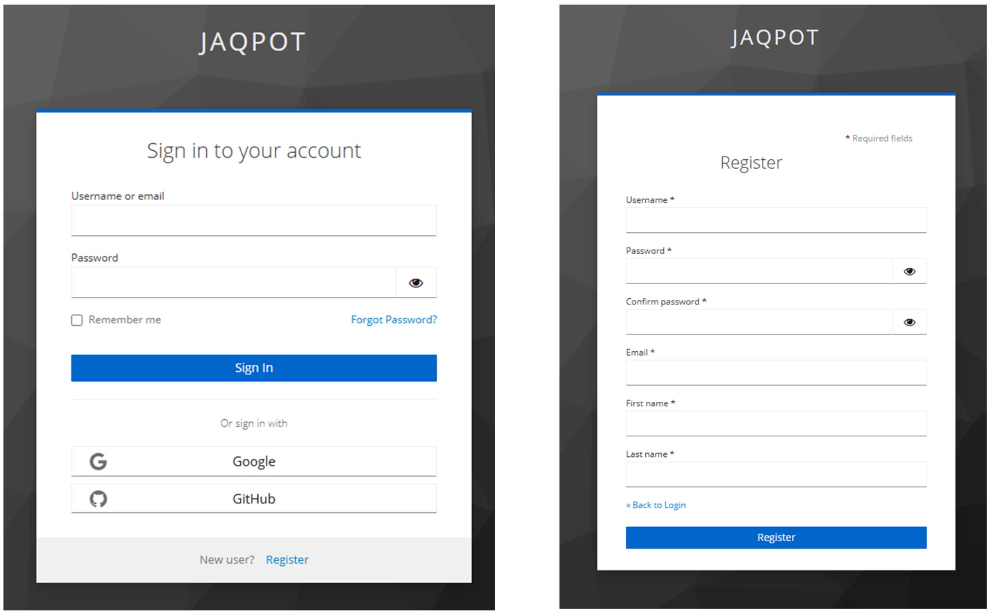

Figure S4. Jaqpot login interface showing the sign-in page (left) and the registration option (right).

The Jaqpot model interface is organized into four tabs: Description (initial), Features, Predict, and Metrics. The Description tab summarizes the model scope and usage (**Figure S5**), while detailed information on input and output variables is provided in the Features tab (**Figure S6**). In the Metrics tab, see **Figure S7**, end-users can examine model performance on the train, test and validation sets.

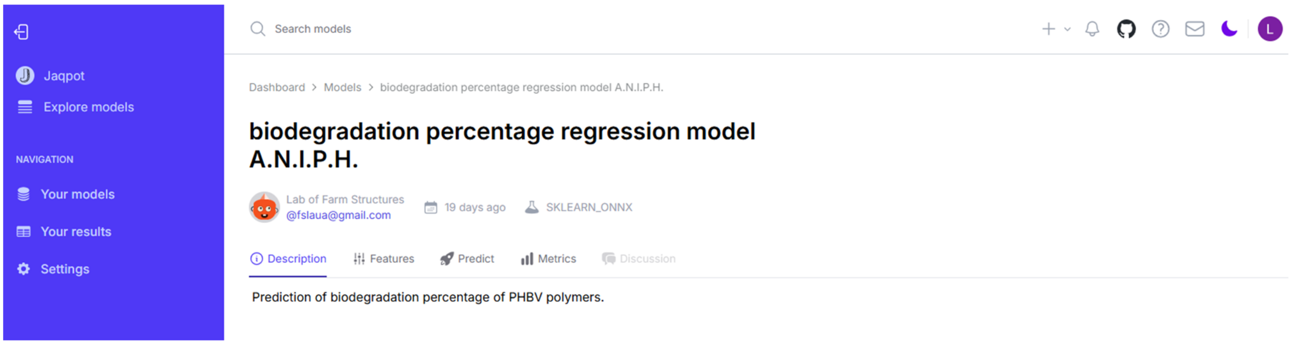

Figure S5. “Description” tab of the prediction model.

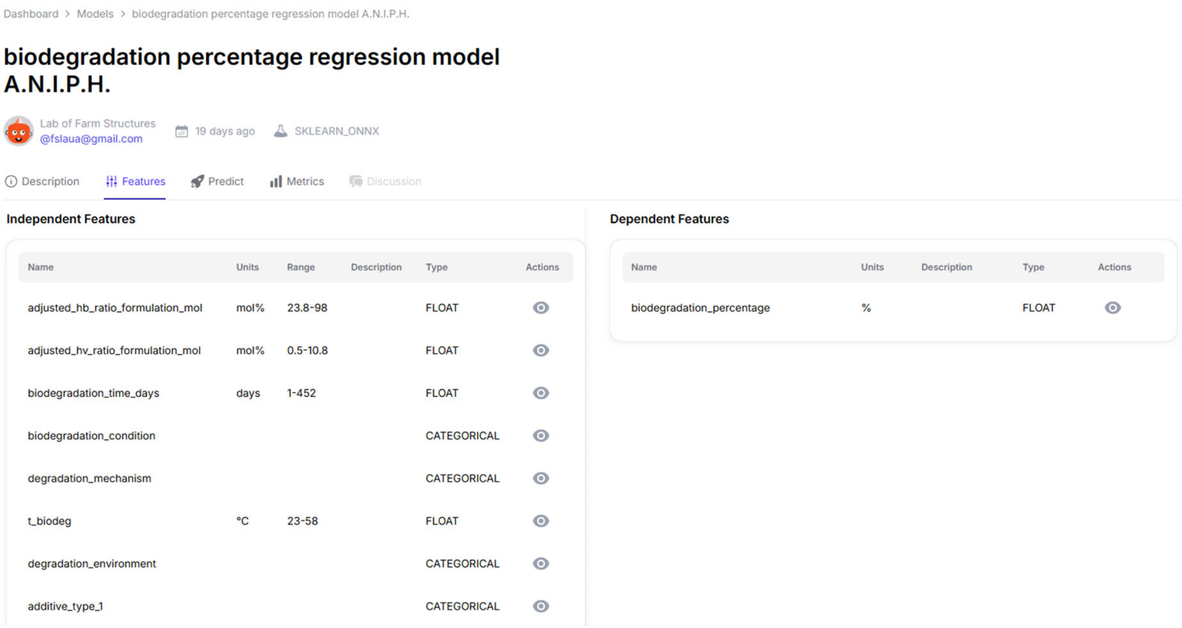

Figure S6. "Features" tab of the prediction model.

In the Predict tab (**Figure S8**), end-users can generate model predictions by either entering a single instance of the input variables (numerical features as individual values and categorical features selected from dropdown lists) or by uploading a CSV file using the provided template. Predictions are obtained by pressing the "Submit" button. The predicted values are displayed in the UI and can be exported as a CSV file. The user is also notified when the entered feature values fall outside the model's applicability domain and should therefore be interpreted with caution.

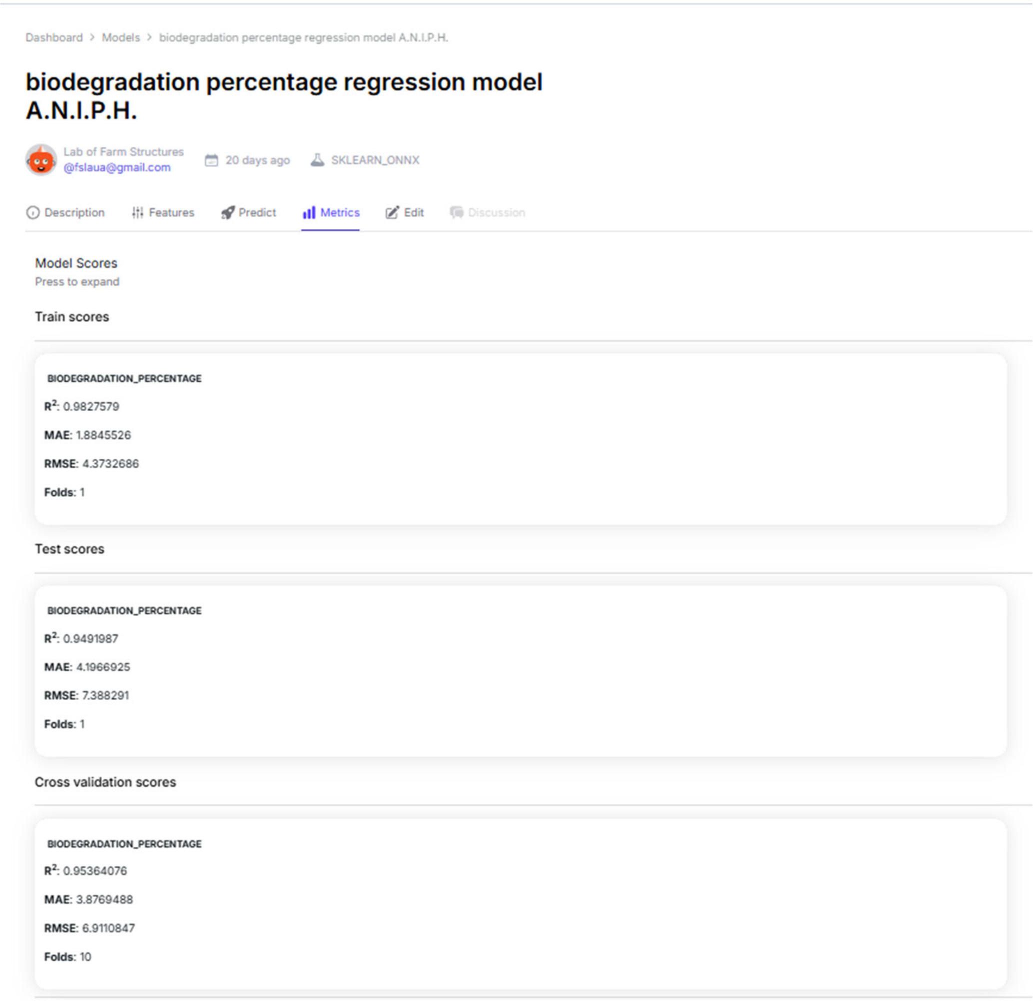

Figure S7. "Metrics" tab of the prediction model.

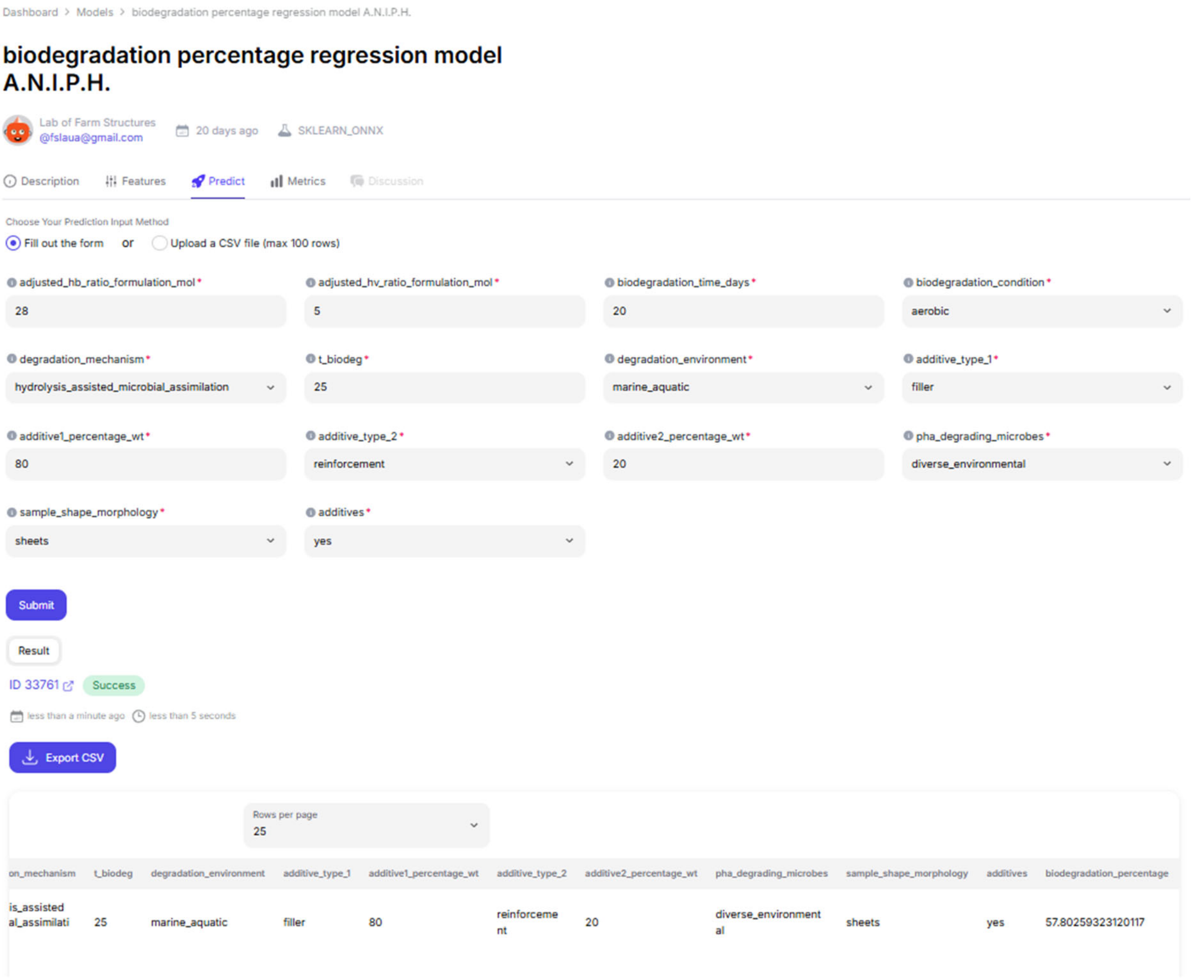

Figure S8. "Predict" tab of the prediction model.
